# Measuring subjective complaints of attention and performance failures - development and psychometric validation in tinnitus of the self-assessment scale APSA

**DOI:** 10.1186/1477-7525-11-86

**Published:** 2013-05-29

**Authors:** Ulli Simone Bankstahl, Roman Görtelmeyer

**Affiliations:** 1 Merz Pharmaceuticals GmbH Global R&D, Frankfurt, Germany; 2 Faculty of Social Sciences, Clinical and Biological Psychology, Universität Mannheim, Mannheim, Germany

**Keywords:** Attention, Performance, Self rating scale, Validity, Reliability

## Abstract

**Background:**

There is a need for a validated self-assessment questionnaire for cognitive impairment in subjects reporting subjective tinnitus. The objective was to develop a patient-reported outcome measure.

**Methods:**

This was a prospective, non-interventional, multicultural study. The 30-item “Attention and Performance Self-Assessment Scale” (APSA) was linguistically validated in Germany, Mexico and USA and was analyzed for content and structure. The analysis included descriptive statistics of baseline data, item characteristics, test-retest reliability (intra-class correlation coefficients, ICC), definition of internal consistency (Cronbach’ s alpha), and explorative and confirmatory factor analysis to define the structure of the scale. Correlations with various tinnitus scales and subscales from the Hospital Anxiety and Depression Scale (HADS) were done to estimate convergent validity.

**Results:**

The data for 211 subjects aged 30 through 60 years, (mean= 48.5 years, SD= 8.3) with mild to moderate tinnitus (mean Tinnitus Handicap Inventory-12 (THI-12) total score 11.2, SD= 5.3) were analyzed. The majority of subjects had sub-clinical scores for anxiety and depression (HADS below 11 points). Sequential principal factor analyses of the APSA resulted in a subscale which included 20 (APS20) of the original 30 items and two correlated subscales (AP-F1, AP-F2) defined by 9 items each. Both factor solutions were confirmed by confirmatory factor analysis. Test retest reliability of the APS20, AP-F1 and AP-F2 (ICC ≥ 0.87) and internal consistency (Cronbach’s alpha ≥ 0.89) are high. APS20 correlated moderately high with HADS (depression: 0.54; anxiety: 0.62) and THI-12 total (0.52). In a few cases, AP-F2 correlated higher than AP-F1 with other scales (e.g. HADS-depression with AP-F1: only 0.46, but AP-F2: 0.59).

**Conclusions:**

APS20, AP-F1, and AP-F2 have good psychometrical properties. The scales will add value to the assessment of cognitive aspects of quality of life and mental health in the population with subjective tinnitus. The subscales AP-F1 and AP-F2 may be helpful for detecting specific cognitive failures and may be sensitive to different interventional effects.

## Background

Tinnitus is not only exceedingly frustrating and annoying, disturbs sleep, interferes with quiet activities and is often associated with hearing loss but patients are also known to suffer other discernible functional impairment. Results regarding performance in psychological tests are ambiguous although it seems that tinnitus may have impact on attentional capacities [1,2]; others report that in objective cognitive tasks tinnitus patients performed almost as well as people without hearing problems or tinnitus [3]. Nevertheless, in one study patients reported more cognitive failures than the control group in the Cognitive Failures Questionnaire [4]. The Cognitive Failures Questionnaire was poorly correlated with functional impairment such as hearing loss, interference with sleep or quiet activities, but moderately correlated with anxiety. The authors conclude that “the most reasonable interpretation of cognitive inefficiency in tinnitus concerns the control of attention and especially the inhibition of attention to task irrelevant activity” [3]. This mirrors other research that found that attention deficits in the performance of tasks in daily life are frequently observed in tinnitus patients [5,6]. It was clear therefore, that any assessment of treatment outcome in subjective tinnitus should include failures in attention and everyday slips and lapses as viewed by the patients themselves, in order to measure an important aspect of their quality of life and mental health [7].

We aimed to develop a self-assessment questionnaire which would allow the scaling of cognitive failures and mishaps to serve as an outcome variable in a clinical developmental program in subjective tinnitus. We reviewed relevant scales and tests for suitability for this purpose. No scale was found to fit our requirements sufficiently [4,8-10]. The questionnaires we analyzed either had item concepts which were too broad or covered larger problems such as the ability to stay awake during a task. In some cases, it was felt that the items might not be sufficiently sensitive to detect change after intervention. Therefore we decided to develop and validate a scale based on psychological principles including concepts which have been more or less explicitly reported by persons suffering from subjective tinnitus (For example as reported in papers [5,6]). The present paper reports the process of item selection, development and analysis of reliability and validity of the new Attention and Performance Self-Assessment Scale in adults with a clinical diagnosis of tinnitus from three countries.

## Materials and methods

### Item pool compilation and adaptation

As a first step, 99 items were collected from various sources found in the literature of studies on attention and cognitive performance [4,5,8-10]. The selection of items was done with the intention of generating an inter-culturally equivalent scale. Therefore the items were to address more or less general aspects of a person’s ability to concentrate and to perform daily living activities. Both are aspects of quality of life and health [7].

Items were selected that addressed attention, concentration, disorientation, memory, continuity, distractibility, cognitive control, cognitive-emotional, mistakes, attribution and symptoms. As far as possible, we selected questions related to daily situations and that were neutral in terms of gender and education level. Items were selected that reflected cognitive mishaps and failures that were commonly recognized as such by the subject, rather than items reflecting specific situational or cultural factors. Soon after starting the collection of questions, we had the impression that specific failures might be age-related. After deleting duplicates and items with similar wording we checked the resulting item pool for face validity and further reduced it to 56 German language items, harmonized for better readability and response options. The items of this pool were tested in 44 German-speaking subjects, 4 of whom were tinnitus sufferers. The volunteers were encouraged to comment on the clarity of the questions and response options. Following their feedback some items and response options were changed. After this process, the final item pool comprised 30 items, each with a five-point response scale and a recall period of 4 weeks. The response options were: never, rarely, sometimes, often and always, with never as 0 and always as 4. The 30-item questionnaire was translated and then backward translated into 6 languages including US English and Mexican Spanish. This was followed by cognitive debriefing interviews with tinnitus patients, a clinician review of each language version and a harmonization meeting. All versions, including the German version, underwent final linguistic reconciliation using the US English edition as the master version [11].

### Study design

This was a prospective, multicultural, non-interventional, observational study in adult subjects with a clinical diagnosis of tinnitus. In the USA there were 5 centers: one private practice, 2 clinical research centers and 2 ear, nose and throat (ENT) centers; in Germany there were 2 ENT specialists and in Mexico one ENT and 1 private practice. All data were collected without altering the patient’s tinnitus clinical management. Adults with a clinical diagnosis of subjective tinnitus who received care from a study physician and met the enrollment criteria were invited to partake in the study. Unfortunately it was not possible to perform stratified selection or to match the subjects from the subgroups for gender, age or educational level. The study was approved by relevant institutional review board, IRBs for the US: Quorum Review IRB; for Germany: Ethik-Kommision der Bayerischen Landesärtzekammer; for Mexico: Comité de Ética e Investigación Christus Muguerza del Parque (Chihuahua), and CimByTa, Centro Investigación Médico Biológica y Terapia Avanzada (Guadalajara) prior to subjects’ enrolment at the site. Investigators explained the study processes and procedures to the potential subjects who met the eligibility criteria. subjects were given the opportunity to ask questions and were provided (either by mail or in-person at the clinic) with a written informed consent form to review. If the patient was not interested in participating, clinical management continued as before. All subjects personally signed and dated the IRB approved informed consent form before enrollment. Quality of data was ensured by regular telephone visits as well as by the monitoring visits at the sites conducted during or after completion of the enrolment at the site. Each subject was asked to self-administer the baseline scales at home and to mail the completed questionnaire to the study center. The study center then mailed the follow-up questionnaire to the subject, to be received 10 to 17 days after completing the baseline assessment. The subject was asked to complete the follow-up assessment and to return the questionnaire by mail to the study center within 7 days. The target window for completion of the follow-up questionnaire was 14–28 days after the completion of the baseline questionnaire.

The inclusion criteria were ≥ 18 and ≤ 75 years of age; clinical diagnosis of persistent subjective, uni- or bilateral tinnitus (i.e. tinnitus could not be absent for > 24 hours at a time); present for at least 3 months; willingness and the ability to comply with the protocol and study procedures, including the ability to understand the written and verbal language of the country. The exclusion criteria were: a clinical diagnosis of intermittent or pulsatile tinnitus; tinnitus as a concomitant symptom of an otological/neurological disease; any treatment, disorder or condition that might cause tinnitus to be unstable.

### Assessments

Subjects were asked to provide self-assessments at baseline and 12 – 30 days later as follows:

(1) Demographic information (baseline only) including educational level (as asked by the categories “Less than 10 years of education”,“10 or more years of education without a university degree”, “University degree”) and tinnitus related information such as duration and description of the symptoms.

(2) The questionnaire on complaints of cognitive failures and mishaps (Attention and Performance Self-Assessment, APSA) with the following response options: ‘never’ (0 points), ‘rarely’ (1 point), ‘sometimes’ (2 points), ‘often’ (3 points), or ‘always’ (4 points).

(3) The THI-12 (Tinnitus Handicap Inventory-12 [12-15]), a 12-item self-report questionnaire that contains items to assess tinnitus-related annoyance, anxiety, frustration, loss of control, tinnitus-related impairment of social life, job, and housekeeping activities. Each item is answered ‘often’ (2 points), ‘sometimes’ (1 point), or ‘never’ (0 points), giving a worst possible total of 24 points. For the THI-12 a total score and the three subscores for ‘Emotional reaction’, ‘Social activities and communication’, and ‘Focused attention’ were calculated.

(4) With the tinnitus rating scale (TRS) and the tinnitus severity scale (TSS) subjects evaluate tinnitus loudness/strength, annoyance, impact on life and severity [12]. The TRS has three, and the TSS one 11-point Likert-items with scores from 0 (no impact) to a maximum of 10 (worst possible impact). The TRS and the TSS were used in two different versions that varied by recall time; one refered to the past month; the other to the past week (‘one-week TRS’, TRSw/‘one-week TSS’, TSSw). To compare the psychometric properties of these two versions in the same population, subjects in the USA were randomized to receive either the ‘one month’ or the ‘one week’ version of these questionnaires. In the Mexican and German samples only the ‘week versions’ was used.

(5) The hospital anxiety and depression scale (HADS, [16,17]) is a standard 14 items self-assessment mood scale developed to detect depression and anxiety in a hospital, outpatient, and community-based population. The HADS anxiety scale and the depression scale both ranges from 0 (most positive) to 21 (most negative). None/Mild symptoms is defined with a score of 10 or less and Moderate/Severe is defined as a score of 11 or greater. Standard validated translations were used for our study [18].

### Statistical methods

Subjects eligible according to the inclusion/exclusion criteria and who completed at least one item on the baseline questionnaire were included in the analyses of the baseline data. Subjects who completed at least one item on the follow-up questionnaire and completed the follow-up questionnaire 12–30 days after the baseline questionnaire were included in the follow-up analyses. All data were summarized using standard descriptive statistics (mean and standard deviation for continuous data, frequencies and percentages for categorical data). To detect differences between the country samples one-way ANOVA for country and Mantel-Haenszel Test for categorical data were performed. For the single APSA items means, standard deviations and the difficulty index (defined as the percentage of subjects giving “never” answer) were calculated. Additionally, to quantify the performance of each single item we calculated probability plots with the underlying model assuming that all items contribute to one dimension.

The analysis of the APSA structure and content was begun with an initial exploratory factor analysis (EFA) [19,20] consisting of a principal factor analysis with squared multiple correlations for the prior communality estimates, based on a common factor model. The number of factors was limited to Eigenvalue > 1 and PROMAX rotation of the extracted factors was used. This was conducted with the baseline data from 30 items of the APSA in the total population. Items with final communality (h < 0.40) were eliminated and the EFA was repeated with the remaining items. The final result of the EFA was then tested by means of confirmatory factor analysis (CFA) with the follow-up data from the total population as well as for the US, the Mexican and German samples separately using structural equation modeling [21]. A number of fit indices have been proposed [22,23]. For our purpose (i.e. estimating the goodness of model fit in each country), the chi-squared GFI (Goodness-of-fit index) seemed to be the most appropriate.

The distribution of the resulting factorial scores was characterized by mean, standard deviation (SD), median, minimum and maximum. Internal consistency was evaluated using Cronbach’s coefficient alpha and was considered acceptable if Cronbach’s alpha was 0.70 or greater. Test-retest reliability (r) was evaluated for each scale and sub-scale by computing the intraclass correlation coefficient (ICC) using Shrout-Fleiss reliability with fixed set (3,1) [24]. Test-retest reliability was demonstrated if the ICC coefficient was 0.70 or greater. Minimally detectable change (MDC) is given by MDC = ±1.96 * √2 * SEM, where 1.96 derives from the 95% confidence interval of no change. The standard error of measurement (SEM) is given by SEM = SD * √(1-r) [25]. Convergent/divergent validity was tested with the THI-12, TSS, TRS, and HADS in the total population and each of the countries using Pearson correlation coefficients. To investigate the potential sensitivity to change from baseline to follow-up Pearson correlation coefficients were also calculated between the APSA subscales on the one hand and the THI-12 and TRS total change scores on the other.

We also included a further sample of healthy volunteers in order to estimate sensitivity (true positive/(true positive + false negative)) and specificity (true negative/(true negative + false negative)) of the APSA subscales. We used a linear logistic regression model to calculate a ROC curve to estimate these parameters.

The analyses were conducted using SAS software, Version 9.2 under the Windows operating system (copyright © 2009 SAS Institute Inc). Confirmatory factor analyses were conducted using LISREL software, Version 8.8 under the Windows operating system (copyright © 2006 Scientific Software International, Inc.). Response distribution graphics were generated with jMetrik, version 2.1.

## Results

Overall 338 subjects were enrolled, N = 299 had valid data at baseline, of these, 169 were in the US, 70 in Germany, and 60 in Mexico. The first view of the subject characteristics revealed remarkable differences between the three countries regarding gender, age and educational level distribution. More of the German subjects were male (41/70, 59%) while more of the US (93/169, 55%) and most of the Mexicans (43/60, 72%) were female. In the German sample 57% (40/70) were younger than 50 years, in the US less than one quarter of the sample (41/196) and in Mexico 47% (28/60) were younger than 50. Also the educational level revealed remarkable differences between the countries. For example in the US only 3% (5/169) were in the group of “Less than 10 years of education”, whereas in Mexico this group comprised 57% (34/60) of the total sample there. These criteria were not controlled by equal distribution or any matching rule. Age was only controlled by the predefined range of 18–75 years.

The difference between the country samples was statistically significant for age (ANOVA, p < 0.001), but not for gender and educational level (non-parametrical analysis, non-zero correlation p = 0.0569). Therefore we decided to restrict the sample to those between 30 to 60 years as it was thought that they were likely to be members of the working population with more or less stable socio-psychological conditions. With this selection we hoped to get a less heterogeneous sample with regard to life-conditions. Further, by the exclusion of older persons we hoped to control for the factor aging on cognitive failures and mishaps.

The characteristics of the final analysis set of N = 213 adult subjects are presented in Table 1. The subjects have a mean age of 48.5 years (SD = 8.3), with a mean THI-12 score of 11.2 (SD = 5.3) representing a moderate handicap [12]. The baseline mean for the TRS week version is 14.8 (N = 153, SD = 7.1) and for the month version 16.4 (N = 58, SD = 6.2). The majority of subjects had anxiety and depression scores below 11 points which is the published threshold in both subscales for “indicating probable presence (‘caseness’) of the mood disorder” [16]. On the whole, the Mexican sample had been living with tinnitus for a shorter time (less than one year: 50%) than the German (30.5%) or US sample (16.8%). The majority of subjects reported permanent tinnitus without interruptions (78.1%).

**Table 1:** Characteristics of the final analysis set by country

**Characteristics**		**US**	**Germany**	**Mexico**	**Total**
All subjects at baseline	N	116	59	38	213
Sex,%	Male	52.6	57.6	31.6	50.2
	Female	47.4	42.4	68.4	49.8
Age at baseline (years)	Mean (SD)	50.9 (7.1)	46.0 (8.3)	45.1 (9.4)	48.5 (8.3)
Educational Level in%	Less than 10 years of education	3.5	37.3	47.4	20.9
	10 or more years of education without a university degree	45.6	44.1	26.3	41.7
	University degree	50.9	18.6	26.3	37.4
When did tinnitus start?, in%	<1 year ago	16.8	30.5	50.0	26.7
	>1 to 3 years ago	26.6	18.6	34.2	25.7
	>3 to 5 years ago	13.3	17.0	5.3	12.9
	>5 to 10 years ago	19.5	17.0	5.3	16.2
	>10 years ago	23.9	17.0	5.3	18.6
Is your tinnitus always present? in%	Permanent without interruptions	81.6	81.4	62.2	78.1
	With short breaks (<1 hour)	10.5	3.4	24.3	11.0
	With long breaks (>1 hour)	7.9	15.3	13.5	11.0
THI-12 total at baseline	Mean (SD)	11.3 (5.1)	11.5 (6.0)	10.4 (4.6)	11.2 (5.3)
HADS Depression	Mean (SD)	4.0 (3.2)	6.5 (4.5)	5.8 (3.0)	5.0 (3.7)
HADS Anxiety	Mean (SD)	7.4 (3.5)	9.0 (4.3)	5.6 (3.9)	7.5 (4.0)

### Scale development

In the majority of cases the APSA was completed without the omission of questions. Only item 17 was not answered by 2 out of 213 subjects. The item response characteristics are summarized in Table 2. The item scores ranged from 0 (minimum of scale) through 4 (maximum of scale), except for items 7 and 14 that had a maximum score of 3.

**Table 2:** Descriptive statistics for the APSA items at baseline and subscale assignment

**Item #**	**Description**	**Mean (SD) at Baseline for N = 213**	**“Never”Response (%Subjects)**	**Items used for the APS20 Score**	**Items used for the AP-F1/AP-F2 Score**
1	Do things not intended to	1.37 (1.15)	30.52	APS20	AP-F1
2	Start over when interrupted	1.54 (1.05)	16.43		
3	Sound disturb reading	1.62 (1.09)	16.90	APS20	AP-F2
4	Make mistakes if low effort	1.52 (0.95)	14.62	APS20	AP-F1
5	Concentrate short period	1.60 (1.04)	14.55	APS20	AP-F2
6	Can't express/tip of tongue	1.70 (1.04)	15.02	APS20	AP-F1
7	Keep doing incorrect task	0.57 (0.79)	58.68		
8	Important do tasks well	3.65 (0.71)	1.42		
9	Pressure in ears	1.87 (1.23)	18.31		
10	Difficult follow conversation >1 talking	2.05 (1.18)	10.85	APS20	AP-F2
11	Stressed after work	2.09 (1.06)	7.89		
12	Daydream instead of listen	1.54 (0.92)	13.62	APS20	AP-F2
13	Distracted by sounds when tired	1.77 (1.09)	13.62	APS20	AP-F2
14	No point start task when weighing on mind	1.02 (0.91)	33.80	APS20	
15	Impatient at work	1.59 (0.93)	11.27	APS20	AP-F2
16	Act differently than planned	1.26 (0.93)	23.94	APS20	AP-F1
17	Satisfied with concentration	2.37 (0.96)	4.74		
18	More focused when deadline	2.52 (1.11)	6.57		
19	Sudden forgetfulness	1.66 (0.98)	13.15	APS20	AP-F1
20	Work better when no music	1.67 (1.26)	22.17		
21	Mistake easily when tired	1.37 (0.97)	11.27	APS20	
22	Pain influence work	1.39 (1.08)	26.29		
23	Forget appointments	0.91 (0.95)	41.78	APS20	AP-F1
24	Can’t find things	1.36 (1.10)	26.42	APS20	AP-F1
25	Concentration suffer when tinnitus severe	2.04 (1.11)	12.21		
26	Return home after forgot things	1.18 (1.01)	29.58	APS20	AP-F1
27	Difficult follow conversation talking quickly	1.64 (1.23)	23.58	APS20	AP-F2
28	Read repeatedly	1.31 (1.07)	27.23	APS20	AP-F2
29	Wonder whether use word correctly	1.04 (0.99)	35.68	APS20	AP-F1
30	Mind wandered	1.70 (1.01)	11.27	APS20	AP-F2

(Item 17 not reversed).

**Figure 1:**
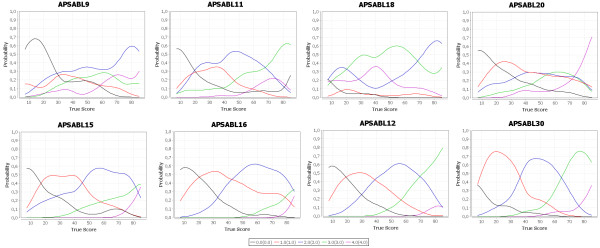
**Response distributions of APSA items.** Response distributions of bad performing items 9, 11, 18 and 20 (upper row) and items with good response properties (lower row).

To further examine the item characteristics we plotted the probability of responses for each item (examples see Figure 1: Response distributions of bad and good performing items) assuming that all items are measuring the same concept. Most of the items showed a good separation of the answering options. Item 17 had good selectivity, but from the wording as well as from the plots it was obvious that the scoring should be reversed if used in a total score. Only the response options of items 9, 11, 18, 20 and 22 did not separate well from each other. Nevertheless we decided to include them in the following factor analyses to see how they would perform.

Further, we wanted to know which of the 30 APSA items were age, gender or education dependent. The results of the general linear model analysis (GLM with factors gender and education level and covariate age using Bonferroni α-correction to control the overall error-type I level of 5%) for the APSA items in the working-age population show that in the USA sample three models are significant. The following effects were observed: Item 15: gender and age; item 16: educational level; item 23: gender and educational level. With all other item we did not observe statistically significant effects of age, gender and/or educational level.

An initial exploratory factor analysis (EFA) with the baseline data of the 30 items in the total analysis set (principal factor analysis, using Eigenvalue >1 criterion for retaining factors) revealed two factors in the inter-item correlation matrix which explained more than 80% of the total variance. Low communality (h < 0.40) was observed for items 8, 9, 18 and 20. The EFA was repeated excluding these 4 items. Again two factors and items with low communality were found. The elimination of items with low communality (h < 0.40) resulted in the exclusion of 10 items in all (items 2, 7, 8, 9, 11, 17, 18, 20, 22 and 25). The last EFA resulted in one common factor explaining 87% of the variance with all remaining 20 items having sufficient high communalities (h ≥ 0.48). This 20-items solution was used to define a score for “attention and performance deficits” which was the sum over these items divided by the number of items answered. For simplicity of communication we call this mean total attention and performance score, APS20. The internal consistency of the APS20 is sufficiently high with a Cronbach’s alpha coefficient of 0.94 (raw score) showing that the items measure the same construct.

Although the results for a one-factor solution and the internal consistency of the APS20 were satisfying we repeated the EFA (principal factor analysis, initial communality: SMC, rotation method: PROMAX) with the baseline data of the 20 items allowing for the extraction of two factors. This was because the second factor’s Eigenvalue had been very close to one and we were curious to know whether information might have been concealed by the single common factor model and the Eigenvalue > 1 criterion. Additional file 1 shows the Rotated Factor Pattern with 2 factors and the variance explained by each factor. The rotated factors were correlated, with r = 0.683. Factor 1, AP-F1, is defined by descriptions of problems with prospective everyday memory including capacity-consuming monitoring; Factor 2, AP-F2, is defined as problems keeping attention focused, necessary to execute tasks in specific situations. For two items (14 and 21) the loadings on the two factors are moderate and quite equal, they will not be used for scaling of the two latent variables in the following analysis.

To confirm the one- and the two-factor models we conducted Confirmatory Factor Analyses (CFA) with follow up visit data using structural equation modeling for each country sample separately and across all countries pooled (see Additional file 2 for the Path diagram of the CFA). The model fit indices GFI for the 1-factor solution were moderately high in all countries (0.58-0.73) and similarly high for the 2-factor solution (0.56-0.71). The lambda value for each item was in an acceptable range; the smallest lambda was 0.53 (item 15: ‘Impatient at work’) in the US sample with the 1-factor solution.

From these results the scaling of the APS20 and the two subscales is justified. The APS20 is calculated from 20 items (1, 3–6, 10, 12–16, 19, 21, 23, 24, 26–30). The 2-factor subscales are computed with 9 items each (AP-F1: 1, 4, 6, 16, 19, 23, 24, 26, and 29; AP-F2: 3, 5, 10, 12, 13, 15, 27, 28, and 30). If 10% or fewer of the items are missing (i.e., 1–2 missing items for the APS20) then the mean score of the non-missing items may be used to estimate the APS20. If more than 10% of the items are missing then the APS20 should be discarded. The scores of the APS20 range from 0 to 4 with higher scores indicating frequent problems with attention and performance.

### Descriptive statistics of the subscales APS20, AP-F1 and AP-F2

The APS20 has an approximately symmetrical distribution with a mean of 1.5 (SD = 0.7; skewness = 0.483) at baseline and 1.5 (SD = 0.7; skewness = 0.363) at the follow up visit (see Table 3 for details of AP-F1 and AP-F2).

**Table 3:** Descriptive statistics, N, Mean (SD) for the APSA scores at baseline

**Criterion**	**Level**	**N**	**APS20**	**AP-F1**	**AP-F2**
Educational level	Less than 10 years of education	44	1.4 (0.8)	1.3 (0.8)	1.6 (0.9)
	10 or more years of education without a university degree	88	1.6 (0.7)	1.5 (0.8)	1.8 (0.8)
	University degree	79	1.3 (0.6)	1.2 (0.7)	1.5 (0.7)
Country	US	116	1.5 (0.7)	1.4 (0.7)	1.7 (0.7)
	Germany	59	1.6 (0.8)	1.5 (0.8)	1.7 (0.9)
	Mexico	37	1.1 (0.7)	0.8 (0.7)	1.4 (0.8)
THI-12 categories	no handicap: THI-12 < 6	36	1.0 (0.6)	1.0 (0.7)	1.1 (0.7)
	mild handicap: 6 ≤ THI-12 < 10	47	1.2 (0.5)	1.1 (0.6)	1.2 (0.5)
	moderate handicap: 10 ≤ THI-12 < 14	63	1.4 (0.6)	1.2 (0.7)	1.7 (0.7)
	severe handicap: THI-12 ≥ 14	63	1.9 (0.7)	1.8 (0.8)	2.2 (0.7)
Total		209	1.5 (0.7)	1.3 (0.8)	1.6 (0.8)

Score ranges from 0 (most positive) to 4 (most negative).

Dependencies of the APS20 score on several variables were analyzed in a general linear model with factors gender, educational level, and country and their interactions, with age as covariate. There was a significant effect of country (p < 0.001), educational level (p = 0.0233) and two significant interactions (gender * educational level, p =0.0043 and country * gender, p = 0.0063).

In the US the mean APS20 score was 1.9, followed by 1.6 in the German sample, and 1.1 in the Mexican sample. Further, subjects from the lowest educational level had the highest mean score with 1.8 and the highest educational level (“university degree”) had the lowest means scores with 1.3. In the US sample the male subgroup had the highest APS20 mean score with 2.3 and in the German sample, females had the highest score with 1.8.

Many items which we excluded due to low communality (h < 0.40) seem to be rather unspecific and were not contentious. Four of the 10 excluded items were easy to agree on (difficulty index < 10%), one item was very difficult (item 7: 58.7%) for the patients.

Two further items are still of interest for further analysis. Item 17 addresses general satisfaction with one’s concentration and item 25 relates cognitive difficulties to the severity of the tinnitus.

### Validity of the subscales APS20, AP-F1 and AP-F2

The discriminant and convergent validity of the APS20, AP-F1 and AP-F2, to the other measures at baseline (THI-12, TRS, TSS and HADS) were analyzed computing Pearson correlation coefficients. An overview of the results is provided in Table 4. The APS20, AP-F1 and AP-F2 were moderately correlated with HADS-A and HADS-D but less correlated with the THI-12 total scale. Interestingly, AP-F1 and AP-F2 behaved differently with regard to THI-12 total score and HADS Depression score. The TRS monthly version item “impact on life” and the TRS monthly version total score (only done in the US, resulting in a smaller sample) were moderately correlated (0.23 ≤ r ≤ 0.48). But there was only weak correlation with the items and the total score of the TRS weekly version and the TSS weekly as well as the monthly version (r ≤ 0.31). This picture changes partially if we look at the inter-scale correlations for AP-F2. As can be seen from Table 4, in many cases AP-F2 correlates higher with the scales than AP-F1 and in a few cases even higher than APS20 does (e.g. correlation with HADS-depression).

**Table 4:** Pearson Correlation Coefficients of APSA scores with other Scales and items at Baseline

**Scale/Item**		**N**	**APS20**	**AP-F1**	**AP-F2**	**Item 17**	**Item 25**
APSA item 17	Satisfied with concentration	209	0.50	0.41	0.52	1.0	0.41
APSA item 25	Conc. suffers when tinnitus severe	209	0.57	0.46	0.61	0.41	1.0
THI-12	Emotional Reaction	206	0.43	0.35	0.48	0.27	0.55
	Social Activities and Communication	206	0.43	0.34	0.49	0.31	0.49
	Focused Attention	206	0.48	0.38	0.53	0.47	0.63
	Overall	206	0.52	0.41	0.58	0.39	0.64
TRS (month)	Loudness	58	0.23	0.23	0.25	0.17	0.30
	Annoyance	58	0.36	0.34	0.37	0.33	0.50
	Impact on life	58	0.48	0.48	0.45	0.16	0.50
	Overall	58	0.43	0.42	0.43	0.25	0.52
TRS (week)	Loudness	149	0.30	0.21	0.34	0.39	0.44
	Annoyance	149	0.31	0.19	0.39	0.33	0.46
	Impact on life	149	0.28	0.18	0.35	0.43	0.49
	Overall	149	0.33	0.21	0.40	0.43	0.51
TSS	TSS (month)	58	0.27	0.26	0.28	0.16	0.38
	TSS (week)	149	0.30	0.21	0.35	0.29	0.47
HADS	HADS Anxiety	206	0.63	0.58	0.59	0.22	0.43
	HADS Depression	206	0.54	0.46	0.55	0.43	0.51

As already mentioned, two of the ten excluded items (items 17 and 25) were used as further validation criteria. The coefficients for Item 17 (satisfaction with concentration), r = 0.50 and item 25 (concentration problems when tinnitus is severe), r = 0.57 are moderately high.

**Figure 2:**
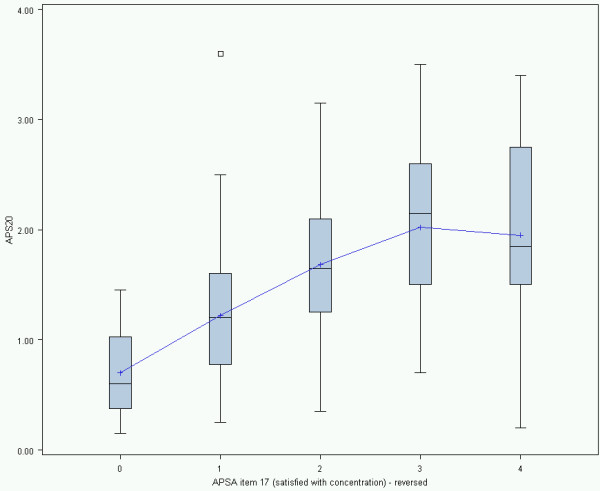
**APS20 across the categories of APSA item 17.** Box-and-Whisker Plot of APS20 across the reversed categories of APSA item 17 (0 = “always”: N = 16; 1 = “often”: N = 92; 2 = “sometimes”: N = 63; 3 = “rarely”: N = 26; 4 = “never”: N = 10).

The box-plot graphs in Figure 2 show that the association between “satisfaction with concentration” (APSA item 17) and APS20 is quite close. Persons who were never or rarely satisfied with their concentration have clearly higher scores on APS20 compared to persons who were often or always satisfied with their concentration. There is a slight drop-down effect of the scores in the category “never” satisfied with concentration, which may be a result of the small number of cases at this end. This association between item 17 scores and APS20, as well as with AP-F1 and AP-F2 is also expressed by the correlation coefficients r = 0.41 (for AP-F1) and r = 0.52 (for AP-F2). This difference can be taken as a further argument for using these two subscales if one wants a differential analysis of cognitive complaints and possible treatment effects.

Not surprisingly, the APSA item 25 (concentration problems when tinnitus is severe) is moderately high correlated with most of the tinnitus scales, whereas item 17 (satisfaction with concentration, reversed scoring) correlates a little less with these scales (see Table 4). Interestingly, item 17 has a low correlation with anxiety (r = 0.22) but a higher correlation with depression (r = 0.42).

For clinical practice the ability of the APSA subscores to discriminate between the severity groups as defined by the THI-12 total score is of considerable importance. For this purpose we used the handicap classification of the THI-12 total score (no/mild/moderate/severe handicap) [12].

**Figure 3:**
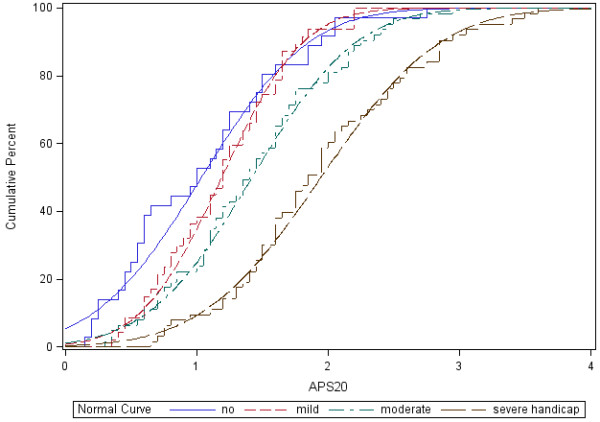
**Separation of APS20 score by tinnitus handicap.** Cumulative distributions for THI-12 categories (no handicap: THI-12 < 6, N = 36; mild handicap: 6 ≤ THI-12 < 10, N =47; moderate handicap: 10 ≤ THI-12 < 14, N =63; severe handicap: THI-12 ≥ 14, N =63).

As can be seen in Figure 3, the cumulative distribution curves of no and mild handicap overlap, whereas the curves for moderate and severe handicap are separated on the APS20 scale. Hence, the APS20 best separates the moderate and the severe tinnitus groups from the other groups. This implies that patients with severe tinnitus handicap very likely show cognitive failures, whereas patients with no or mild handicap are only associated with occasional mishaps.

### Reliability and internal consistency of the subscales APS20, AP-F1 and AP-F2

The APS20 as well as AP-F1 and AP-F2 show very high stability over time (mean duration of retest interval was 17 days (SD: 3.9) from baseline to follow-up, ranging from 12 to 30 days, scores are listed in Additional file 3). In detail, the ICC (3,1) is high for the APS20 (ICC = 0.9), as well as for AP-F1 (ICC = 0.91) and to a slightly smaller but still acceptable degree for AP-F2 (ICC = 0.87). Internal consistency as measured by Cronbach’s alpha coefficient is high for APS20 as well as for AP-F1 and AP-F2 with alpha ≥ 0.89.

### Sensitivity to change and minimally detectable change (MDC)

To estimate sensitivity to change we used the change from baseline to follow-up on the APS20, AP-F1 and AP-F2. As there was no systematic intervention or change in intervention during the observational time, any change could only have been due to the consenting process and explanations by the clinician. We used the difference to baseline in tinnitus measures (TRS and THI-12 total scores) as criteria to estimate the sensitivity to change. As can be seen in Additional file 3, the mean change scores from Baseline were low for the tinnitus scales and nearly zero for the APSA subscales with considerable variability in individual change. The change scores in THI-12 correlated significantly with those in APS20 (r = 0.33), AP-F1 (r = 0.21) and AP-F2(r = 0.41). Also, correlations between change in TRS are significant for APS20 (r = 0.31) and AP-F2 (r = 0.40). The MDC, derived using the sample distribution, variability, and test-retest reliability of the APSA subscales, indicate whether a difference between baseline and a follow up visit represents a true difference in cognitive impairment. The values for MDC are ±0.6 for APS20 and AP-F1, and ±0.8 for AP-F2.

### Sensitivity and specificity

Further, we included a sample of healthy volunteers to estimate sensitivity and specificity of the APSA subscales. The data of this survey from a working-age population (N = 35, age range from 30 to 60 years with APS20 mean= 1.14, SD= 0.37) who had no tinnitus complaints showed no difference compared to the tinnitus subjects who according to THI-12 had no or mild tinnitus handicap (THI-12 ≤ 9) on any APSA subscales. There is good discrimination between healthy volunteers and ‘moderate tinnitus handicap’ (10 ≤ THI-12 < 14) on the APS20 (p = 0.0269) and the AP-F2 (p = 0.0014), as well as healthy volunteers versus ‘severe tinnitus handicap’ (THI-12 ≥ 14) in APS20, AP-F1 and AP-F2 (all p < 0.0001). Therefore we estimated sensitivity and specificity on the no/mild handicap group as the “negative” and the moderate/severe handicap group as the “positive” group. For APS20 we found 87% sensitivity, 46% specificity; for AP-F1: 85% sensitivity, 44% specificity; and for AP-F2: sensitivity of 89%, specificity of 54%.

## Discussion

Complaints of cognitive failures, mainly regarding attention and cognitive performance are frequently reported by tinnitus patients [5,6] but results in objective cognitive tasks are unequivocal. In one study these authors could show that tinnitus patients did not perform differently from healthy persons, matched for intellectual ability, gender and age [3]. Nevertheless attention and concentration may be impaired in tinnitus patients [2]. In order to be able to demonstrate efficacy of a treatment in subjective tinnitus it would be important to demonstrate not only effects on tinnitus scales but additionally to show improvement in cognitive functions which are relevant to performing adequately in daily life. This would help to generate evidence for treatment benefit as attention and cognitive performance are important elements for the patient’s quality of life and mental health [7].

We reviewed several scales from literature to develop a patient-reported outcome measure for a clinical development program of a drug for subjective tinnitus. The concepts and wordings which we found were in many cases not useful for our clinical program. Therefore we adapted and reworded several items. The recall period of four weeks seems to be appropriate as certain failures or mishaps may appear rarely but they are still remembered for a time after the event. For the response options we decided to use a five point scale including zero for “never” and four for “always”. We evaluated the first collection of items in a small sample of healthy volunteers including four persons suffering from tinnitus. From this survey the resulting 30 item questionnaire (APSA) was translated and linguistically validated in several languages [11]. This version was used in the present prospective, non-interventional study in three different countries (USA, Germany, and Mexico) for psychometrical validation in adults with the diagnosis of subjective tinnitus. Since there is no gold-standard for tinnitus measurement, we used several measures that have achieved broad use in these countries.

After recruitment of N = 299 eligible subjects with subjective tinnitus we observed remarkable differences between the country populations regarding age. In order to achieve a less heterogeneous analysis population we decided to reduce the sample to those between 30 and 60 years of age who were most probably members of the working population living in more or less stable socio-psychological conditions.

Overall the means of the single items were between 0.57 and 2.52, the difficulty index (defined as percentage of subjects with “never” answer) varied between 1.42% and 58.68%. The whole scale range (0 – 4) was used except for two items. Some items did not work well according to their response probability plots. Not surprisingly the badly performing items (no. 9, 11, 18, 20, and 22) had low communalities in the initial explorative factor analysis (EFA) and were excluded from further analyses. The final EFA on the remaining 20 items revealed a main common factor explaining 87% of the variance. In the search for further relevant subscales we used PROMAX as rotation procedure and found two latent variables, AP-F1 (prospective everyday memory problems) and AP-F2 (difficulties keeping attention focused). Empirically these two latent variables are correlated indicating links in cognitive performance which are in accordance to psychological theories [26]. From these results we recommend using the 22 items APSA containing the items of the APS20 and the two items 17 and 25 to measure cognitive impairment in subjective tinnitus.

The subscale APS20, as well as AP-F1 and AP-F2 have high internal consistency (≥ 0.89) and stability (≥ 0.87). The convergent validity of the APSA subscales with psychopathological symptoms was only moderate. Interestingly, the subscale AP-F2 showed different correlations with the various baseline data from the other scales. It correlated higher with THI-12 subscale “Focused Attention” than the AP-F1 did. This confirms the construct validity of AP-F2, “difficulties keeping attention focused”. The correlations between the APSA subscales and the tinnitus scales TRS and TSS were low (0.18 ≤ r ≤ 0.48). This indicates that the APSA subscales are measuring different phenomena from the tinnitus scales in our study.

The correlation of APS20 with HADS anxiety (r = 0.63) is in agreement with observations reported for the Cognitive Failures Questionnaire for anxiety: “The highest correlations are between CFQ, Trait Anxiety, and SHHI. This suggests that cognitive failure scores are related to general personality self-descriptions.” [3]. From these results one may conclude that these complaints, expressed in APS20, AP-F1, and AP-F2, seem to be more influenced by self-assessed symptoms of anxiety and depression and less by tinnitus characteristics [27,28].

Our results for sensitivity and specificity show that healthy volunteers and subjects with “no” to “mild tinnitus handicap” have no relevant cognitive impairments. Sensitivity is high with moderate specificity for subjects with “moderate” to “severe tinnitus handicap”. The AP-F2 is 89% sensitive and 54% specific to detect those subjects. The lack of specificity may be considered a limitation. However, subjects with severe tinnitus handicap are not necessarily cognitively impaired by their tinnitus, but may have more emotional problems.

In a non-interventional study sensitivity to change is difficult to estimate. We used the change in tinnitus severity (defined as THI-12 total and TRS total change scores) as indicator for change. Sensitivity to change is moderate for APS20 and AP-F2; further studies are needed to make robust estimates of sensitivity of change. The results of the minimally detectable change test suggest that the APS20 should decrease by at least 0.6 points or 12 item responses by one grade, before any improvement beyond reproducibility noise can be detected.

The strength of our study is that we developed a questionnaire addressing many cognitive functions which are relevant for adequate performance of daily life activities, including attention and memory, planned activities, and retrospective and prospective memory. Other cognitive domains may also be of clinical importance in subjective tinnitus. The APSA is indicated for persons who are capable of introspection and are aware of cognitive failures and mishaps which they are able to remember when answering the questionnaire. It may also be indicated in persons with mild cognitive deficiencies or impairments but is probably less sensitive for cases of severe cognitive impairments. Our results are limited to persons with confirmed subjective tinnitus aged between 30 and 60 years. Another limitation is that we did not include subjects with more prominent cognitive impairments. Further studies will be necessary to extend the scope to other cognitive domains and age groups, also objective cognitive tests to validate the APSA against test performance are necessary. For further inter-cultural validity of the APSA, larger, preferably stratified samples may be necessary, as the study site selection performed here was not representative for the tinnitus populations in these countries.

## Conclusions

The APSA subscales, APS20, AP-F1, and AP-F2 have good psychometrical properties. They are stable, and internally consistent. AP-F1 is dominantly defined by problems with prospective memory problems whereas the AP-F2 is defined by difficulties keeping attention focused. These two subscales may be helpful for detecting differential cognitive processes and specific interventional effects. The APSA is indicated for people who are capable of remembering and evaluating their own cognitive failures and mishaps in daily life. The subscales are less correlated to tinnitus characteristics than to symptoms of depression and anxiety. The questionnaire is a promising tool with added value in assessing cognitive aspects of quality of life and mental health in the adult population suffering from subjective tinnitus.

For copies of the APSA please send an email to scales@merz.de.

## Competing interests

Merz supported this research. The authors are employees of the company sponsoring this research. The authors declare no conflict of interest.

## Authors’ contributions

USB planned the design and methods, analyzed the data of the psychometric validation study, and worked on the concept of the present data analysis. RG planned the design and methods, worked out the analysis concept and carried out the present data analysis. Both authors contributed to and have approved the final manuscript.

## Supplementary Material

Additional file 1 **Rotated Factor Pattern of EFA with 2 Factors.** Exploratory Factor Analysis (EFA) (principal factor analysis, initial communality: SMC, rotation method: PROMAX) with the baseline data of the 20 items allowing for extraction of two factors. The Table shows the rotated factor pattern (Standardized Regression Coefficients) and the variance explained by each factor. The rotated factors are correlated by r = 0.683. Factor 1, AP-F1, seems to reflect more general memory problems, Factor 2, AP-F2, comprises perceptual or attentional problems in specific situations. Click here for file

Additional file 2 **Path diagram of CFA. **Path model for the 2-factor solution in the Confirmatory Factor Analysis (CFA) using follow-up data with all countries pooled. GFI = 0.82, Model AIC = 393.69. Click here for file

Additional file 3 **Descriptive statistics of questionnaire scores.** N, Mean, SD, Min and Max are reported for THI-12, APSA subscales and TRS for Baseline and Change from Baseline to Follow-up. Click here for file
