# Supplementary material for: Measuring subjective complaints of attention and performance failures - development and psychometric validation in tinnitus of the self-assessment scale APSA
Source: Health Qual Life Outcomes. 2013 May 29;11:86. doi: 10.1186/1477-7525-11-86 (PMC3674948; doi:10.1186/1477-7525-11-86)
Supplement: Additional file 1 — Rotated Factor Pattern of EFA with 2 Factors. Exploratory Factor Analysis (EFA) (principal factor analysis, initial communality: SMC, rotation method: PROMAX) with the baseline data of the 20 items allowing for extraction of two factors. The Table shows the rotated factor pattern (Standardized Regression Coefficients) and the variance explained by each factor. The rotated factors are correlated by r = 0.683. Factor 1, AP-F1, seems to reflect more general memory problems, Factor 2, AP-F2, comprises perceptual or attentional problems in specific situations. [file 1477-7525-11-86-S1.pdf]

Additional file 1 –Rotated Factor Pattern of EFA with 2 Factors

Exploratory Factor Analysis (EFA) (principal factor analysis, initial communality: SMC, rotation method: PROMAX) with the baseline data of the 20 items allowing for extraction of two factors. The Table shows the rotated factor pattern (Standardized Regression Coefficients) and the variance explained by each factor. The rotated factors are correlated by r = 0.683. Factor 1, AP-F1, seems to reflect more general memory problems, Factor 2, AP-F2, comprises perceptual or attentional problems in specific situations.

Rotated Factor Pattern (Standardized Regression Coefficients)

	FACTOR1	FACTOR2
APSA item 1	**0.73363**	0.01064
APSA item 3	0.17329	**0.53176**
APSA item 4	**0.47604**	0.25992
APSA item 5	0.19776	**0.56600**
APSA item 6	**0.64870**	0.11185
APSA item 10	-0.03692	**0.73471**
APSA item 12	0.25229	**0.50106**
APSA item 13	0.03859	**0.68838**
APSA item 14	0.31594	0.37447
APSA item 15	0.29863	**0.42132**
APSA item 16	**0.59319**	0.13656
APSA item 19	**0.77252**	-0.01073
APSA item 21	0.35517	0.40684
APSA item 23	**0.58875**	0.14460
APSA item 24	**0.73652**	0.03795
APSA item 26	**0.74108**	-0.01210
APSA item 27	-0.08788	**0.77246**
APSA item 28	0.11021	**0.69174**
APSA item 29	**0.49044**	0.31043
APSA item 30	0.25774	**0.57497**
Variance Explained by Each Factor Eliminating Other Factors:	2.318763	2.118615

Bold: higher loading resulting in mapping to this factor
