# Supplementary material for: Measuring subjective complaints of attention and performance failures - development and psychometric validation in tinnitus of the self-assessment scale APSA
Source: Health Qual Life Outcomes. 2013 May 29;11:86. doi: 10.1186/1477-7525-11-86 (PMC3674948; doi:10.1186/1477-7525-11-86)

Additional file 2 – Path diagram of CFA

Path model for the 2-factor solution in the Confirmatory Factor Analysis (CFA) using follow-up data with all countries pooled. GFI=0.82, Model AIC = 93.69

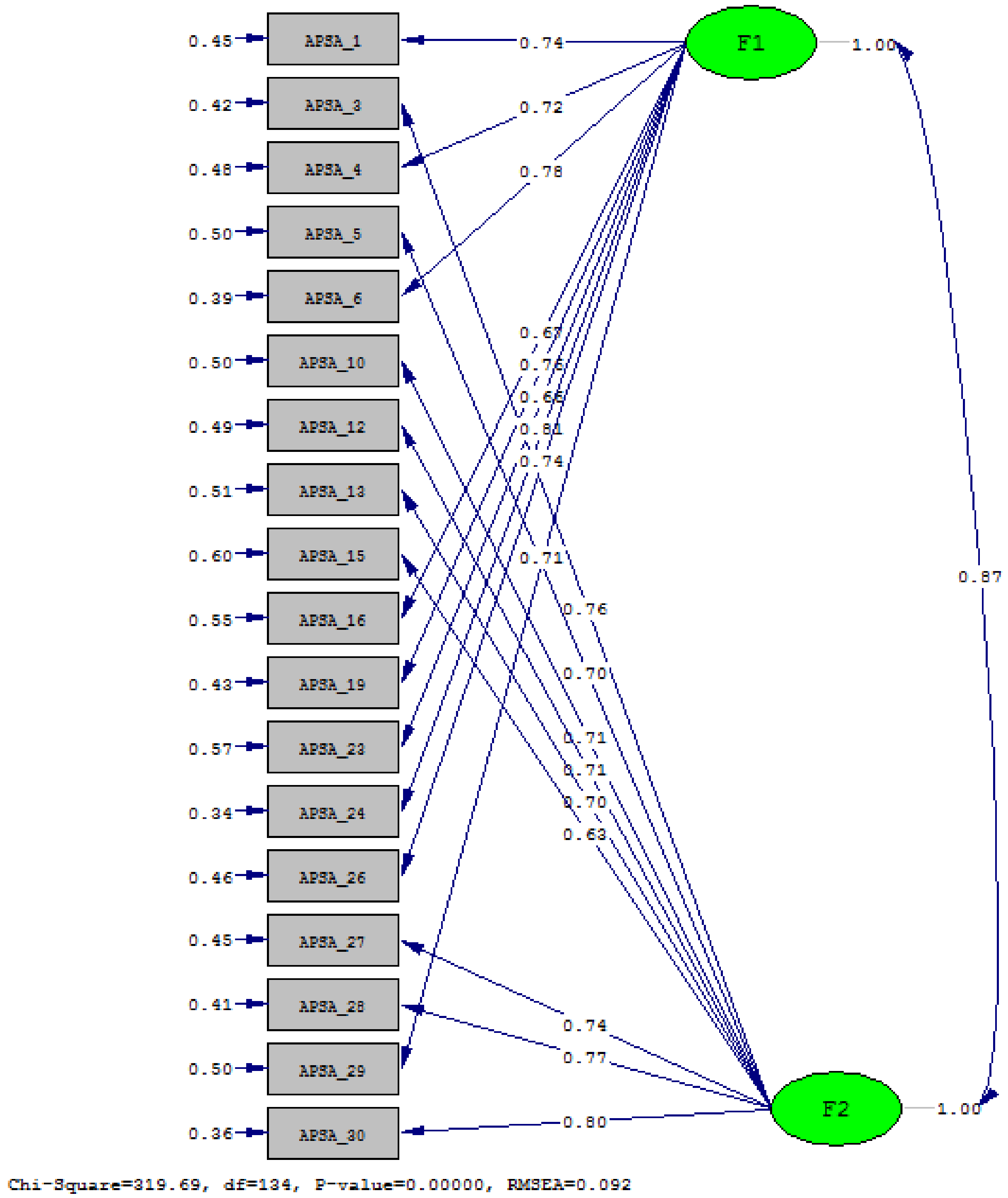

Supplement: Additional file 2 — Path diagram of CFA. Path model for the 2-factor solution in the Confirmatory Factor Analysis (CFA) using follow-up data with all countries pooled. GFI = 0.82, Model AIC = 393.69. [file 1477-7525-11-86-S2.pdf]
