# Supplementary material for: Measuring subjective complaints of attention and performance failures - development and psychometric validation in tinnitus of the self-assessment scale APSA
Source: Health Qual Life Outcomes. 2013 May 29;11:86. doi: 10.1186/1477-7525-11-86 (PMC3674948; doi:10.1186/1477-7525-11-86)
Supplement: Additional file 3 — Descriptive statistics of questionnaire scores. N, Mean, SD, Min and Max are reported for THI-12, APSA subscales and TRS for Baseline and Change from Baseline to Follow-up. [file 1477-7525-11-86-S3.pdf]

Descriptive Statistics of scale scores

Scores at Baseline:

Variable	N	Mean	Std Dev	Minimum	Maximum
APS20	209	1.46	0.71	0.15	3.60
AP-F1	209	1.32	0.76	0.00	3.67
AP-F2	209	1.63	0.78	0.11	3.89
THI-12 total	211	11.18	5.26	1.00	24.00
TRS total month	58	16.41	6.16	4.00	30.00
TRS total week	153	14.82	7.14	0.00	30.00

Change from Baseline to Follow-up (score from Baseline minus score from Follow-up):

Variable	N	Mean	Std Dev	Minimum	Maximum
APS20	166	-0.02	0.31	-0.95	0.65
AP-F1	166	-0.01	0.32	-1.00	0.89
AP-F2	166	-0.01	0.39	-1.22	1.11
THI-12 total	175	0.62	2.62	-6.00	15.00
TRS total month	45	0.71	2.57	-7.00	6.00
TRS total week	129	-0.29	4.13	-14.00	15.00
